# Corrigendum: Hidden non-Fermi liquid behavior caused by magnetic phase transition in Ni-doped Ba-122 pnictides

**DOI:** 10.1038/srep14153

**Published:** 2015-09-22

**Authors:** Seokbae Lee, Ki-Young Choi, Eilho Jung, Seulki Roh, Soohyeon Shin, Tuson Park, Jungseek Hwang

Scientific Reports 5: Article number: 1215610.1038/srep12156; published online: 07 17 2015; updated: 09 22 2015

The original version of this Article contained a typographical error in the spelling of the author Seulki Roh, which was incorrectly given as Seulki Rho. This has now been corrected in both the PDF and HTML versions of the Article.

